# Impact of vascular density on pancreatic islet viability: a computational study

**DOI:** 10.3389/fphys.2025.1707026

**Published:** 2025-11-26

**Authors:** Gerardo J. Félix-Martínez, Lizbeth de la Cruz, Daniel Whisman, J. Rafael Godínez-Fernández

**Affiliations:** 1 Department of Electrical Engineering, Universidad Autónoma Metropolitana, México City, Mexico; 2 Department of Biological Sciences, Idaho State University, Pocatello, ID, United States

**Keywords:** pancreatic islet, vasculature, oxygenation, computational model, interactive tool

## Abstract

**Introduction:**

Pancreatic islets are densely vascularized clusters of cells that secrete insulin, glucagon, and somatostatin, hormones essential to glucose metabolism. The viability and function of islet cells rely on the availability of oxygen and nutrients supplied by islet capillaries. In this work, we developed a computational model of vascularized pancreatic islets, aiming to estimate the proper level of vascularization to ensure cell viability.

**Methods:**

The simulated islets were reconstructed from experimental data of human and mouse, with capillaries generated using a pathfinding algorithm. The number of capillaries required to maintain islet cell viability was determined by analyzing oxygen gradients resulting from cell consumption at 6- and 20-mM glucose concentrations and a varying number of capillaries.

**Results:**

Our simulations in human and mouse models suggest that >100 capillaries (i.e., vascular density > 5.9%) are required to maintain >96% of cells in a viable state at 6- and 20-mM glucose. From this percentage of viable cells, >75% of cells are in functional state (*P*_*O2*_ > 10 mmHg) at 6 mM glucose while it reduces to ∼50% at 20 mM glucose.

**Discussion:**

These models represent an advancement in computational models available to study islet physiology by incorporating the vascular system to enable more accurate, predictive, and physiologically relevant simulations in health, disease, and bioengineering approaches where changes in the islet vasculature are relevant. To support the broad application of the model, we provide a user-friendly computational tool where the viability of islet cells can be estimated based on a given number of capillaries, islet size and glucose level.

## 1 Introduction

Pancreatic islets are composed mainly of α, β and δ-cells, endocrine cells responsible for the secretion of glucagon, insulin and somatostatin, respectively - key hormones for the regulation of blood glucose. Islets are densely vascularized, as indicated by reports showing that even though islets represent only ∼2% of pancreatic tissue, they receive close to 20% of total pancreatic blood flow (Rorsman and Ashcroft, 2018; Burganova et al., 2021). The dense capillary network of pancreatic islets supports their endocrine function by enabling rapid blood glucose sensing and efficient hormone release into bloodstream, while also meeting their oxygen and nutrients needs and removing metabolic waste. Each islet is typically supplied by one to five arterioles, which branch into a dense network of fenestrated capillaries that facilitate efficient exchange of nutrients, hormones and oxygen. The islet capillary network drains through venules into the insulo-acinar portal system (Gonçalves and Almaça, 2021).

While the vascular system within pancreatic islets has long been recognized as essential for 1) facilitating the exchange of oxygen, nutrients, and waste to support cell metabolism and 2) enabling islet cells to sense blood glucose levels, which then triggers appropriate hormone secretion, emerging evidence suggests that the capillary network within islets is highly regulated and its regulation plays a critical role in hormone secretion. From this perspective, regulatory mechanisms impact the islet capillary network, changing local blood flow, which can impact nutrient sensing by islet cells and, consequently, hormone secretion. This regulation may be influenced by neural input affecting the capillary-pericyte complex within the islet or pathological changes directly associated with endothelial cells (Almaça and Caicedo, 2020). Alterations in the vascular system have been linked to the development of insulin resistance (Okajima et al., 2022), obesity, and type 1 and type 2 diabetes (Burganova et al., 2021; Canzano et al. 2019; Brissova et al., 2015; Almaça et al., 2020). These findings underscore the importance of the vascular system in islet physiology as a key component in understanding endocrine function in health and metabolic disease.

Despite the importance of their vascular system, when isolated for experimentation, islets are excised from the surrounding tissue and therefore, from the vasculature. In these conditions, islets are dependent on the slow diffusion of peripheral oxygen, which presents a challenge to their viability, as both experiments and computational models have shown (Félix-Martínez et al., 2024; Komatsu et al., 2017). Similarly, when isolated for transplantation purposes, islets also experience severe difficulties maintaining cell viability in the absence of their capillary network. To overcome these limitations, different approaches have been tested, such as the engineering of microvascular structures to enhance the revascularization of transplanted islets (Shang et al., 2025; Li et al., 2025). However, the quantitative relationship between capillary number, oxygen availability, and cell survival remains poorly characterized.

Computational models have become essential to study and understand the collective behavior of α, β, and δ-cells within pancreatic islets. Early models focused on coupled β-cells and have progressed to multicellular islet models that incorporate experimentally derived geometries and both electrical and paracrine pathways (for a recent review see ref. (Félix-Martínez and Godínez-Fernández, 2023)). Despite this progress, most models omit key features that shape islet function such as the capillary network, a key player for the rapid exchange of hormones and nutrients. On the other hand, other models have focused on detailed hemodynamic features at the lumen of blood vessels, although ignoring the surrounding cellular environment (Tuveri et al., 2022; Wang et al., 2014; Petkova et al., 2003). Furthermore, due to their computational costs, such models are restricted to small vascular segments which makes them still impractical for a comprehensive pancreatic islet model. Based on this, and aiming to lay the foundations for integrating vascular networks into computational models of pancreatic islets, here we develop a computational model that integrates, for the first time, the islet vasculature in multicellular models derived from experimental data, in contrast to previous islet models focused on inter-islet communication (electric and/or paracrine) and oxygenation in avascular islets. The proposed approach allows us to quantitatively estimate the degree of vascularization needed to provide mice and human islets with proper oxygenation, to quantify the relationship between capillary number, oxygen availability, and cell survival in human and mouse islet models, and to analyze how islet size and glucose concentration impact oxygen gradients and cell viability at different levels of vascularization. In addition, we provide with an easy-to-use computational tool designed to facilitate the widespread use of the model, allowing users to estimate the viability of islet cells based on factors such as the number of capillaries, the size of the islet, and the glucose level.

## 2 Materials and methods

### 2.1 Conceptual model

This model simulates how a varying number of capillaries could be distributed within the complex environment of pancreatic islets, which contain α, β, and δ-cells. Starting from detailed experimental data on cell positions and types from human and mice islets, with varying sizes, the model first reconstructs the three-dimensional layout of the islet by placing cells in space according to their observed locations and sizes (Section 2.2). These cells are treated as fixed obstacles within a virtual grid that represents the islet tissue.

Capillary paths are initially created as simple routes that avoid the space occupied by islet cells (Section 2.3). Then, the model allows capillary cells to grow and reshape dynamically within the available space by gradually rearranging small volume units within the grid (Section 2.4). This rearrangement is driven by a process that mimics physical principles, such as how cells prefer to stick together and maintain certain sizes, while also incorporating randomness to explore different possible shapes and avoid getting stuck in less optimal arrangements.

Afterwards, oxygen distribution is simulated across the tissue, accounting for how oxygen delivered by capillaries acting as sources, diffuses and is consumed by the cells with varying consumption rates depending on cell type and glucose concentration (Section 2.5). This approach allows us to determine which cells are viable, hypoxic, or non-viable based on the oxygen availability. By integrating these elements, the model captures how the islet vascular network supply oxygen within the spatial constraints of the islet, providing insight into the relationship between capillary density, cell viability, and islet size under different conditions.

### 2.2 Computational reconstruction of pancreatic islets

The reconstruction of six human and mice islets was performed in IsletLab, following the method previously outlined (Félix-Martínez et al., 2022; Félix-Martínez, 2022). Islet reconstruction was based on experimental data generously shared by Hoang et al. (Hoang et al., 2016; Hoang et al., 2014), composed of 3D architectures of human and mouse islets including cell positions and cell types, obtained from individual islets isolated from 3-month-old mice and from human pancreases from 40–60-year-old donors, provided by the Gift of Hope Organ and Tissue Donor Network in Chicago. In human islets, α, β, and δ-cells were included while in mice islets, only α and β were considered. Experimental data used for the reconstruction process was obtained through confocal microscopy and immunofluorescence. In summary, the reconstruction algorithm begins by creating an initial islet model, placing spherical cells at the experimental coordinates, and assigning radii randomly selected from distributions derived experimentally. The initial configuration is then evaluated by calculating the number of overlapping cells. During the optimization process, a cell is randomly chosen in each iteration, and new center coordinates and radii are assigned. The overlap count was recalculated and compared with the smallest count previously recorded. If there is a reduction, the change is accepted; otherwise, it might be accepted or rejected with a decreasing probability to avoid local minimum entrapment. This iterative optimization progresses until convergence, or a predefined criterion is achieved. Details about the simulated islets are given in Table 1.

**TABLE 1:** Dimensions of the simulated islet environments and total number of islet cells (*N*_*cells*_ *)*. *N*α, *N*_β_ and *N*δ are the number of α, β and δ-cells. Grid voxels were cubic with side length 1 μm. Note that δ-cells were not considered in mice models.

Human islet	*X* (μm)	*Y* (μm)	*Z* (μm)	*N*_*cells*_	*N*_α_	*N*_β_	*N*_*δ*_
1	227	208	164	583	148	316	119
2	309	364	196	2,252	427	1,461	364
3	340	388	225	3,323	1,082	1,523	617
4	359	389	237	3,516	961	2,209	346
5	282	263	219	2,084	642	1,168	274
6	313	307	230	2,841	830	1,355	656

Mice islet	*X* (μm)	*Y* (μm)	*Z* (μm)	*N*_*cells*_	*N*_α_	*N*_β_	*N*_*δ*_
1	172	170	241	570	34	536	-
2	274	215	272	1,229	35	1,194	-
3	392	312	172	2,097	135	1,962	-
4	351	237	266	2,596	469	2,127	-
5	339	344	243	3,195	219	2,976	-
6	396	397	198	4,005	286	3,719	-

### 2.3 Initial spatial configuration of the simulated islets

**FIGURE 1:**
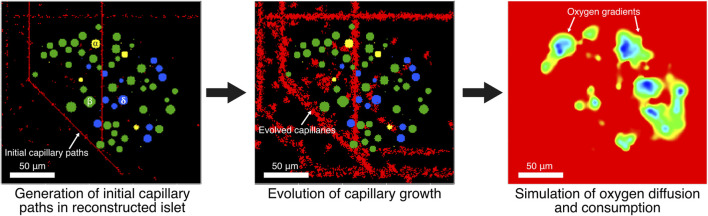
Initial capillary paths are generated in computationally reconstructed pancreatic islets. Then, radial growth of capillaries is evolved. Oxygen gradients are simulated at 6- and 20-mM glucose using capillaries as oxygen sources while endocrine cells consume oxygen in a glucose-dependent manner.

The reconstructed islets were mapped onto a 3D grid where each voxel corresponds to a discrete cubic spatial unit with a 1 μm side length. The grid dimensions, shown in Table 1, were determined based on the bounding box of the islet, with an additional buffer space (30 μm) added to ensure sufficient room for capillary generation. α, β and δ-cells were mapped onto the grid as obstacles for the capillary generation algorithm. The voxels within the sphere were marked with unique identifiers corresponding to the cell type and treated as fixed cell voxels to ensure that capillary paths would avoid intersecting cellular regions. Capillary paths were generated based on the 3D grid representation of the islet environment. Initial capillary paths were generated using the Best-First Search algorithm, a heuristic-based pathfinding method. The algorithm was implemented on the 3D grid to find collision-free paths between randomly selected start and end points as follows. Random start and end points were chosen within the grid faces. For instance, for *x*-direction paths, the start point was on one face of the grid, and the end point was on the opposite face. Similarly, paths were generated in the *y*- and *z*-directions. For each direction, 100 paths were generated (300 total). The initial grid, including islet cells and the initial capillary paths were imported into CompuCell3D 4.6 (Swat et al., 2012) to evolve the radial growth of capillaries (see Figure 1). Simulations with a variable number of capillaries were performed by randomly selecting the required number of capillaries from the 300 capillaries generated.

### 2.4 Evolution of islet capillaries

Capillary shape and size evolved from the initial capillary paths with length $L_{c0}$ , determined from the grid dimensions of the reconstructed islets, using the Glazier-Graner-Hogeweg (GGH) method (Swat et al., 2012), where system evolution follows energy minimization of the Hamiltonian:

$$H=\sum_{\left(i,j\right)}J_{\tau \left(\sigma_{i}\right)\tau \left(\sigma_{j}\right)}\left(1-\delta_{\sigma_{i}\sigma_{j}}\right)+\sum_{i}\lambda_{\tau \left(\sigma_{i}\right)}{\left(v_{i}-V_{\tau \left(\sigma_{i}\right)}\right)}^{2}$$ (1)

where σᵢ denotes the cell ID at lattice site *i*, $\tau \left(\sigma_{i}\right)$ the cell type, i.e., medium (*m*), α, β, δ or capillary cell (*c)*, $J_{\tau \left(\sigma_{i}\right)\tau \left(\sigma_{j}\right)}$ the adhesion energy between cell types, $v_{i}$ the current cell volume, $V_{\tau \left(\sigma_{i}\right)}$ the target volume, and $\lambda_{\tau \left(\sigma_{i}\right)}$ the volume constraint strength. The Kronecker delta ( $\delta_{\sigma_{i}\sigma_{j}}$ ) ensures that only interfaces between different cells contribute to the boundary energy. Parameters used to evolve the islet capillaries are given in Table 2. Through successive Monte Carlo updates, the GGH algorithm minimizes the total Hamiltonian (Equation 1) by probabilistically testing and accepting or rejecting voxel copy attempts between neighboring lattice sites according to the Metropolis criterion. In each update, a voxel and one of its neighbors are randomly selected, and the voxel may adopt the neighbor’s identity if this change reduces the system’s total energy, or with a small probability even if it slightly increases it, thus allowing stochastic exploration of possible configurations. Randomness in voxel and neighbor selection, as well as in the probabilistic acceptance of copy attempts, ensures unbiased sampling of the lattice and prevents the system from becoming trapped in local energy minima. This iterative process gradually drives the system toward a lower-energy state, where interfacial and volume constraint energies are minimized. As a result, capillary voxels rearrange to grow, elongate, and adapt their morphology within the available extracellular space defined by the fixed endocrine cells.

**TABLE 2:** Parameters of the model of capillary evolution (Equation 1). Contact energies (in arbitrary energy units, a.e.u.) defines adhesion between cell types (lower values indicate stronger adhesion), target volume specifies the preferred capillary size (depends on the average capillary radius and a predefined length $L_{c0}$ calculated from the islet size), volume constraint strength penalizes deviations from that size, and model temperature controls the stochasticity of voxel rearrangements.

Parameter	Value	Description
$J_{cm}$	70	Contact energy capillary/medium (a.e.u)
$J_{\alpha m}$	80	Contact energy α-cell/medium (a.e.u)
$J_{\beta m}$	80	Contact energy β-cell/medium (a.e.u)
$J_{\delta m}$	80	Contact energy *δ*-cell/medium (a.e.u)
$J_{c\alpha }$	10	Contact energy capillary/α-cell (a.e.u)
$J_{c\beta }$	10	Contact energy capillary/β-cell (a.e.u)
$J_{c\delta }$	10	Contact energy capillary/*δ*-cell (a.e.u)
$J_{cc}$	1E3	Contact energy capillary/capillary (a.e.u)
$V_{c}$	$2\pi r_{c}^{2}L_{c0}$	Target volume of capillaries (μm^3^)
*r*_*c*_	3.5	Average capillary radius (μm)
$\lambda_{c}$	1E3	Capillary volume constraint strength (μm^3^)
*T*	10	Model temperature (a.u.)

### 2.5 Oxygen diffusion and consumption

Oxygen diffusion was represented as a continuous field on the 3D grid representing the islet environment, governed by a time-dependent partial differential equation of the form:

$$\frac{\partial P_{O_{2}}}{\partial t}=D\nabla^{2}P_{O_{2}}-\lambda_{\sigma_{i}\in \left\{\alpha ,\beta ,\delta \right\}}\left(x\right)P_{O_{2}}+S_{\sigma_{i}=v}\left(x\right)$$ (2)

as implemented in CompuCell3D (Swat et al., 2012), where *P*_*O2*_ is the local partial pressure of oxygen, *D* is the diffusion coefficient of oxygen in islet tissue (2.1 × 10^−9^ m^2^/s (Félix-Martínez et al., 2024)), $\lambda_{\sigma_{i}\in \left\{\alpha ,\beta ,\delta \right\}}\left(x\right)$ is the cell dependent local oxygen consumption rate, and $S_{\sigma_{i}=v}\left(x\right)$ is the oxygen source term, valid only for the voxels occupied by blood vessel cells (*v*). The oxygen diffusion-reaction equation (Equation 2) is solved using a finite-difference discretization on the same grid employed for the capillary growth, where oxygen concentration values are updated at each time step according to local diffusion fluxes, consumption and source terms until steady-state oxygen distributions are reached.

Oxygen was assumed to diffuse uniformly throughout the islet, with a constant oxygen concentration of 7.02 μM along capillary voxels, equivalent to an oxygen partial pressure of 30 mmHg (assuming a solubility of 2.34 × 10^−4^ mol/(mmHg · m^3^)), in the range of the experimental reported partial pressure in native mice islets measured using Clark microelectrodes (Carlsson et al., 1998; Carlsson et al., 2003; Olsson et al., 2011). Only α, β and δ-cells were assumed to consume oxygen with consumption rates per voxel estimated from our previous work (Félix-Martínez et al., 2024) for two glucose concentrations (6 and 20 mM, see Table 3). Functional, hypoxic, and non-viable cell states were determined based the local cell oxygen concentrations and the thresholds adopted in previous works (Félix-Martínez et al., 2024; Buchwald, 2009; Buchwald, 2011; Papas et al., 2019), that is, functional: *PO*_*2*_ > 10 mmHg, hypoxic: 0.45 mmHg *< PO*_*2*_ < 10 mmHg and non-viable: *PO*_*2*_ < 0.45 mmHg, determined by the average *PO*_*2*_ in the cells’ voxels. The population of cells composed of hypoxic and functional cells was identified as viable cells.

**TABLE 3:** Oxygen consumption rates for α, β and δ-cells at 6 and 20 mM glucose (used in Equation 2). Estimated from (9).

Parameter	Glucose (mM)	Value (μM/ms)
$\lambda_{\alpha }$	6	0.1313
$\lambda_{\beta }$	6	0.1810
$\lambda_{\delta }$	6	0.1170
$\lambda_{\alpha }$	20	0.2040
$\lambda_{\beta }$	20	0.4850
$\lambda_{\delta }$	20	0.2450

**FIGURE 2:**
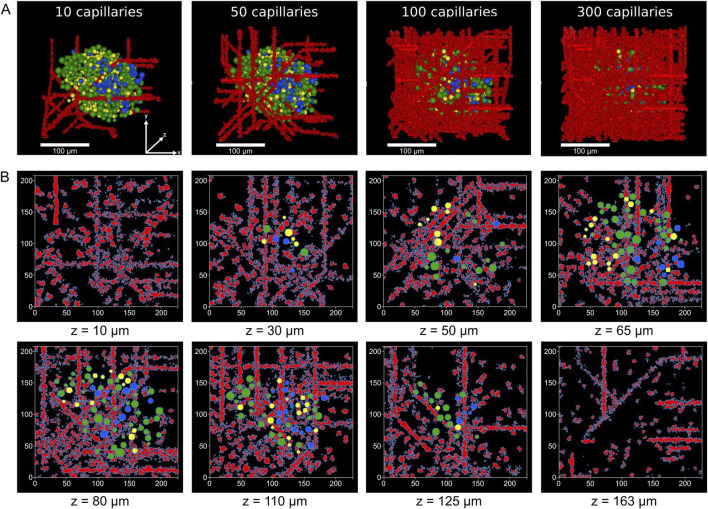
Vascularization of reconstructed human islets. **(A)** 3D renderings of the model of islet 1 with increasing numbers of capillaries, showing spatial relationships between blood vessels (red) and α (yellow), β (green) and δ-cells (blue). All islets shown share the same coordinate axes. **(B)** Selected z-plane slices from islet 1 with 100 capillaries illustrating the distribution of the vascular network and islet cells across different depths (z = 10–163 μm). Capillary voxels are shown in red, while endocrine cells are color-coded as in **(A)**.

**FIGURE 3:**
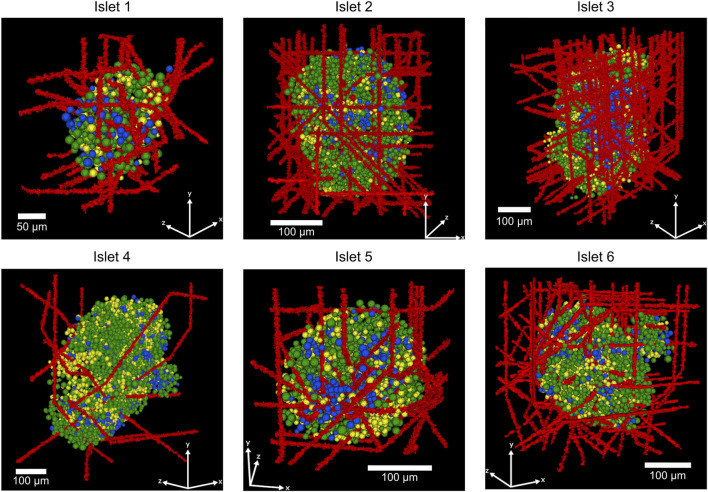
Representative 3D-reconstructed human islets showing the spatial organization of α, β, and δ-cells and a varying number of capillaries (islet 1: 25 capillaries, islet 2: 50 capillaries, islet 3: 100 capillaries, islet 4: 10 capillaries, islet 5: 25 capillaries, islet 6: 100 capillaries). Red structures represent blood vessels (capillaries), green cells indicate insulin-producing β-cells, yellow cells show glucagon-producing α-cells, and blue cells represent somatostatin-producing δ-cells.

Examples of the vascularized human islets generated are shown in Figure 2, where islet 1 is presented with varying number of capillaries. Similarly, in Figure 3, six vascularized human islets are shown, illustrating the heterogeneity in islet size, shape, cellular composition, and complexity of the vascular network. Animations showing a three-dimensional representation of the vascularized islets, both from humans and mice, can be found in the associated repository (https://github.com/gjfelix/VascularizedIsletsModel).

### 2.6 Model of cell viability

We used a modified sigmoidal model to characterize the relationship between the proportion of viable cells (*V*), the number of capillaries (*N*_*cap*_), and the number of cells (*N*_*cells*_). The model takes the form:

$$V=V_{\min}+\frac{V_{\max}-V_{\min}}{1+{\left(\frac{C_{50}}{N_{cap}}\right)}^{n_{C}}{\left(\frac{N_{50}}{N_{cells}}\right)}^{n_{N}}}$$ (3)

where *V*_*min*_ and *V*_*max*_ represent the lower and upper bounds of viability proportion, respectively, *C*_*50*_ is the capillary density at which viability is half-maximal, *N*_*50*_ is the cell number at which viability is half-maximal, *n*_*C*_ and *n*_*N*_ are coefficients determining the steepness of the viability response to *N*_*cap*_ and *N*_*cells*_, respectively.

The model was fitted for the whole islet under G6 (6 mM) and G20 (20 mM) glucose conditions using nonlinear least-squares regression, with capillary positions held constant to isolate oxygenation effects. Goodness-of-fit was assessed via the determination coefficient, *R* ^*2*^, and the mean squared error. This approach allowed us to quantify the effects of increased oxygen consumption due to an increase in glucose concentration and predict viability loss due to an increase in islet size.

### 2.7 Computational details

Simulations were performed on a PC with an Intel Core i7 processor (3.8 GHz × 16) and 64 GB of RAM memory. The configuration of the initial 3D grids, correlation and statistical analyses, and the fitting of the viability models were performed in Python 3.11. Capillary growth and simulations of oxygen diffusion and consumption were performed in CompuCell3D 4.6 (Swat et al., 2012). Code related to this article and model files can be obtained from the associated repository (https://github.com/gjfelix/VascularizedIsletsModel).

### 2.8 Animal model

Male C57BL/6 WT mice were purchased from the Jackson Laboratory (12–16 weeks, RRID:IMSR_JAX: 000,664). Animals were kept in an animal facility under controlled conditions and were given standard chow and water *ad libitum*. The animal handling protocol was approved by the Idaho State University Institutional Animal Care and Use Committee.

### 2.9 Immunohistochemistry

Pancreas tissues were collected from mice and then fixed in a 4% paraformaldehyde solution. Samples were dehydrated using increasing glucose gradients (10%, 20%, and 30%) and then placed in an embedding medium for frozen tissue specimens. Then, the tissue was sliced into 200 µm sections using a cryostat machine and stored at 4 °C for immunohistochemistry application for up to 3 weeks.

Pancreas slices were incubated in a blocking solution consisting of 0.25% Triton X-100% and 3% Bovine Serum Albumin in 1X PBS for 24 h on a rocker at 4 °C. After this period, slices were transferred to the incubation with primary antibodies in blocking solution in a rocker at 4 °C for 48 h. Following this incubation, samples were washed with 1X PBS for 20 min in a rocker to remove residual antibodies. The wash step was repeated for a total of four washes. Then, slices were incubated in secondary antibodies in 1XPBS for 24 h at 4 °C, covered with aluminum foil to prevent light penetration. After this incubation, samples were washed four times with 1X PBS in a rocker for 20 min. For primary antibodies, Insulin–mouse (Santa Cruz, #sc-377071) and CD31-goat (R&D Systems, AF3628) were used to detect pancreatic islets and blood vessels respectively. Donkey anti-mouse 488 (Invitrogen, A32766) and donkey anti-goat 647 (Invitrogen, A32849) were used as secondary antibodies.

After immunohistochemistry, a clearing protocol was performed to assess intact islets within the pancreatic tissue. Samples were transferred to an increasing concentration of ethanol solution. The concentrations used were 25%, 50%, 75%, then two sequential wells with 100% ethanol. Samples were placed in each concentration for 5 min, then transferred to the next. Then, samples were transferred to ethyl cinnamate, first for 3 min then in a new tube with clean cinnamate for 10 min. Finally, samples were mounted in a chamber and scanned using a confocal FV1000 microscope in the ISU Molecular Research Core Facility.

### 2.10 Vasculature analysis in experimental and simulated data

For experimental data, confocal images were analyzed using Fiji (Schindelin et al., 2012) and a stack of ten images of sequential Z sections in the middle of the islet (images every 1 µm). For vessels diameter measurements, data were collected using the plot profile tool by measuring the distance between peaks (peaks correspond to the walls of the vessels). For the vascular density measurements, a mask associated with the vessel area was created for the entire islet section, ensuring that only the area within the islet was included. Then, the area of the whole islet was measured. Density was calculated by dividing the area of the vessels by the total islet area and then multiplying by 100.

For simulated vasculature in the model, the capillary diameters were also measured using Fiji (Schindelin et al., 2012) by randomly selecting capillaries from the middle z slice from the six reconstructed mice and human islets. Diameter vessels and vascular density were quantified following the same protocol described from experimental data. Statistical analyses were performed in GraphPad Prism version 10 (GraphPad Software, Boston, Massachusetts, United States). Normality tests were performed using the Shapiro-Wilks test. Differences between the diameters of the simulated and experimental capillaries were analyzed using the Kruskal–Wallis test and the Dunn’s *post hoc* test for pairwise comparisons.

## 3 Results

### 3.1 Comparison of the vasculature of simulated islets with experimental data

**FIGURE 4:**
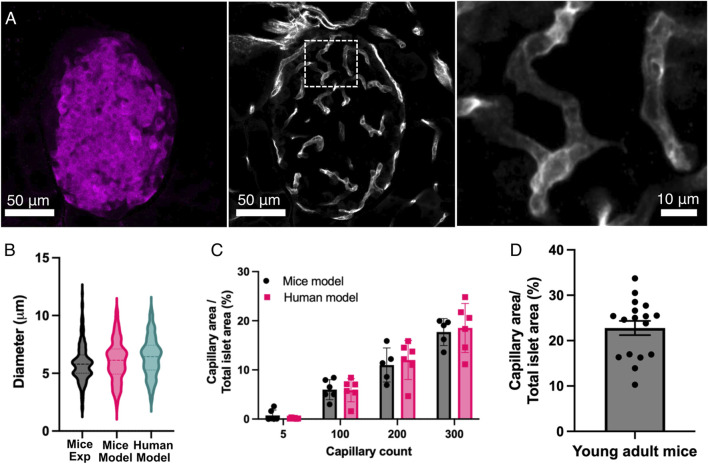
Comparison of the computational model with experimental data. **(A)** This panel shows representative images of the vascular system within the pancreatic islets. The pancreas was taken from young adult mice. The left panel shows an insulin mark that defines the area of the pancreatic islet. The middle panel shows the vascular system (capillaries) marked with CD31 from the islet shown in the left panel. The right panel shows an expansion of the capillaries within the islet, as seen in the middle panel (white box). **(B)** Comparison of the diameter of the capillaries within pancreatic islets in the mice (n = 176) and human (n = 289) models and experimental data from mice islets (n = 177). **(C)** Relationship between capillary count and vascular density (capillary area/total islet area) from the computational models. Individual data points are plotted alongside means ± SEM (n = 6 islets in both human and mice islet models). **(D)** Vascular density (capillary area/total islet area) from 17 pancreatic islets in young mice. Experimental data were collected from 177 vessels across 17 islets from three animals.

To validate our model, we first characterized the vasculature by comparing the diameters of the simulated capillaries with experimental data from young mice islets. Analysis of the islet vascular network was restricted to the islet area determined by the insulin mark as shown in Figure 4A. Figure 4B shows the comparison of distributions of capillary diameters between the modeled and experimental islets. While diameters of capillaries of mice islets showed an average value of 5.86 μm ± 1.62 μm (n = 177), the simulated capillaries in human and mice islets had an average diameter of 6.44 ± 1.69 μm (n = 289) and 6.09 ± 1.75 μm (n = 176), respectively. In both cases, diameters measured were consistent with physiological dimensions observed in pancreatic microvasculature (Gonçalves and Almaça, 2021; Canzano et al., 2019; Cohrs et al., 2017; Jansson and Carlsson, 2019; Diez et al., 2017; Almaça et al., 2018).

Then, we examined the relationship between capillary count and vascular density in the simulated islets (Figure 4C), a measure commonly used to characterize the degree of islet vascularization from experimentally derived images. Human and mice Islets with minimal vascularization (5 capillaries) showed low capillary area percentages (<1%), while moderately vascularized islets (100 capillaries) exhibited a vascular density of 5.87% ± 2.37% in humans and 5.98% ± 2.04% in mice. Higher capillary counts (i.e. 200 and 300 capillaries) in mice islets resulted in vascular densities of 11.00% ± 3.47% and 17.70 ± 2.74, respectively. In human islets, 200 and 300 capillaries corresponded to a vascular density of 12.00% ± 3.98% and 18.53% ± 4.98%, respectively. Statistical analyses revealed significant differences in vascular density between all the capillary count groups (p < 0.05). Measurements of vascular density in experimental mice islets showed a mean capillary density of 22.8% ± 1.2% (Figure 4D), which aligned most closely with our model predictions for both humans and mice at the higher capillary counts (200–300 capillaries).

These findings are consistent with experimental data from rodent and primate pancreatic islets (Canzano et al., 2019; Li et al., 2025; Diez et al., 2017; Chen et al., 2021). By successfully reproducing the architectural features of islet microvasculature, our modeling approach provides a quantitative framework for investigating how variations in capillary number or density could impact islet function considering oxygen availability and consumption, islet size and glucose concentration.

### 3.2 Impact of capillary number and islet size on oxygen supply and cell viability

**FIGURE 5:**
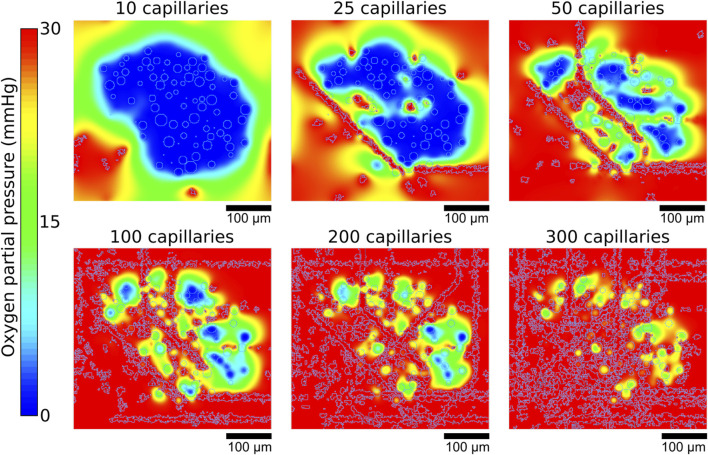
Representative example of the oxygen gradients produced depending on the number of capillaries. Gradients shown correspond to the central z slices of the simulated islet 1.

Simulations of oxygenation in human and mice islets showed that a higher number of capillaries resulted in higher average oxygen partial pressures, as illustrated in Figure 5 showing data from islet 1. With 10 capillaries, the islet core showed uniform low oxygen levels (showed in blue). As capillary density increases, the oxygen distribution pattern transitions from a continuous hypoxic core to fragmented areas of varying oxygenation, with most tissue regions achieving higher oxygen tensions (showed in red). This demonstrates how increased vascularization enhances oxygen perfusion throughout the islet tissue, potentially supporting improved cell viability and function.

**FIGURE 6:**
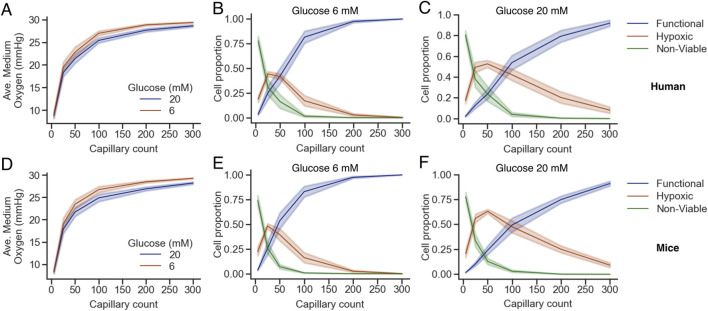
Oxygen availability and cell viability depend on capillary density and glucose concentration in human **(A–C)** and mice **(D–F)** islets. **(A,D)** Average oxygen concentration in the islet medium as a function of capillary count for two glucose concentrations (6 mM, brown; 20 mM, blue). **(B,C,E,F)** Proportions of functional (blue), hypoxic (orange), and non-viable (green) cells across capillary densities at glucose 6 mM **(B,E)** and 20 mM **(C,F)**. Shaded areas represent the standard error of the mean across simulations. Note that B,C and E,F share the same legend.

The average medium oxygen is shown in Figures 6A, D for human and mice islets, respectively. In both cases, a mild but noticeable decrease in the average medium oxygen was observed when glucose was raised from 6 to 20 mM, which can be explained by the increased consumption by the endocrine islet cells at 20 mM glucose. Complementarily, the proportion of functional, hypoxic and viable cells for G6 and G20 are shown in Figures 6B, C, E, F, respectively. As the number of capillaries increased, the proportion of functional cells rose steeply, particularly between 5 and 100 capillaries, while hypoxic and non-viable populations declined correspondingly. Between 5 and 50 capillaries, there was an increase in the proportion of hypoxic cells as non-viable cells transitioned towards the functional range. The main difference at G20, in comparison to G6, is that the proportion of hypoxic cells increased beyond 0.5 at G20 at the expense of a less steep increase in the proportion of functional cells as a function of the number of capillaries. Notably, the percentage of non-viable cells dropped sharply with even modest increases in vascularization, reaching near-zero levels beyond approximately 100 capillaries at G6 (1.55% ± 2.54% and 0.80% ± 0.84% in humans and mice islets, respectively), which translate to >97% and >99% of viable cells (i.e., hypoxic + functional) in human and mice islets, respectively. In contrast, at G20, the percentage of non-viable cells was slightly higher (3.96% ± 4.65% and 3.03% ± 2.70% in humans and mice islets, respectively), with the corresponding reduction in the percentage of viable cells (>96% in both cases). These results highlight the global dependence of islet viability on capillary density and reinforce the notion that insufficient vascularization is a critical driver of hypoxia and non-viability in the islet microenvironment.

**FIGURE 7:**
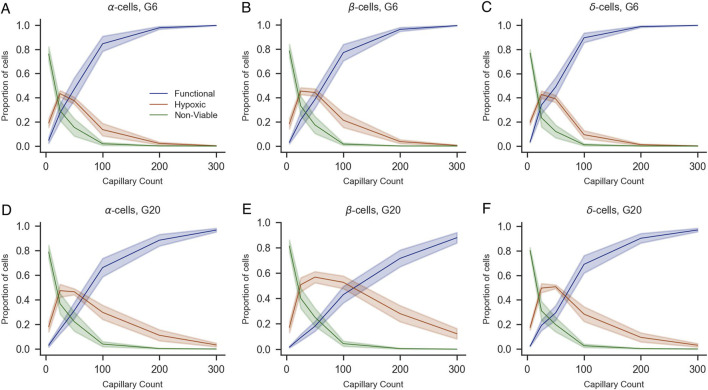
Impact of capillary density on the viability of α-, β-, and δ-cells of human islets under varying glucose concentrations (G6 and G20). Proportions of functional (blue), hypoxic (orange), and non-viable (green) α-cells **(A,D)**, β-cells **(B,E)**, and δ-cells **(C,F)** are shown as a function of capillary count under 6 mM **(A–C)** and 20 mM glucose conditions **(D–F)**. Shaded areas represent standard error of the mean across simulations. All panels share the same legend shown in **(A)**.

**FIGURE 8:**
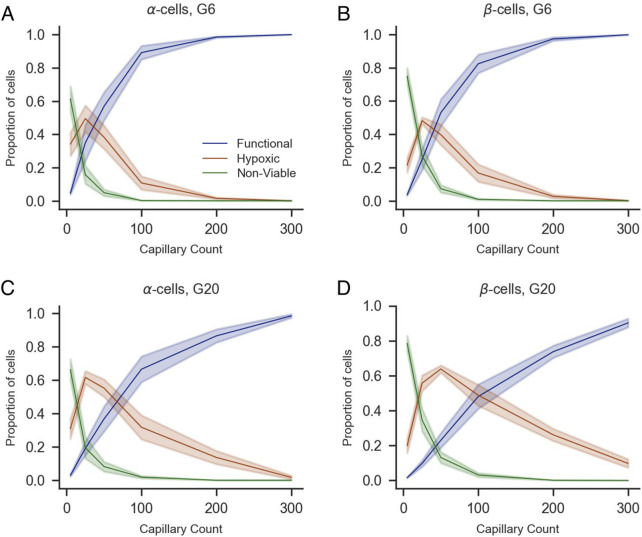
Impact of capillary density on the viability of α- and β-cells of mice islets under varying glucose concentrations (G6 and G20). Proportions of functional (blue), hypoxic (orange), and non-viable (green) α-cells **(A,C)**, β-cells **(B,D)** are shown as a function of capillary count under 6 mM **(A,B)** and 20 mM glucose conditions **(C,D)**. Shaded areas represent standard error of the mean across simulations. All panels share the same legend shown in **(A)**.

To complement this analysis, we quantified the proportions of functional, hypoxic, and non-viable cells by population (α, β, and δ-cells for human islets and α and β for mice islets) under G6 and G20 conditions with increasing numbers of capillaries (Figures 7, 8). The overall trends mirrored those observed in the whole islet cell population: a higher number of capillaries was associated with a marked increase in the proportion of functional cells and a concurrent decrease in both hypoxic and non-viable populations. These effects were most pronounced between 0 and 100 capillaries, after which the proportions tended to stabilize. Notably, at G20, the proportion of functional β-cells exhibited a more gradual increase as the number of capillaries increased, which prevented the totality of β-cells from reaching the functional state. Instead, a non-negligible proportion of β-cells remained hypoxic even with 300 capillaries, in contrast to the same scenario at G6, where practically 100% of β-cells were in the functional range with 300 capillaries. The same effects of the increased oxygen consumption at G20 were noticeable to a lesser extent on the populations of α and δ-cells, the latter only evaluated in human islets.

**FIGURE 9:**
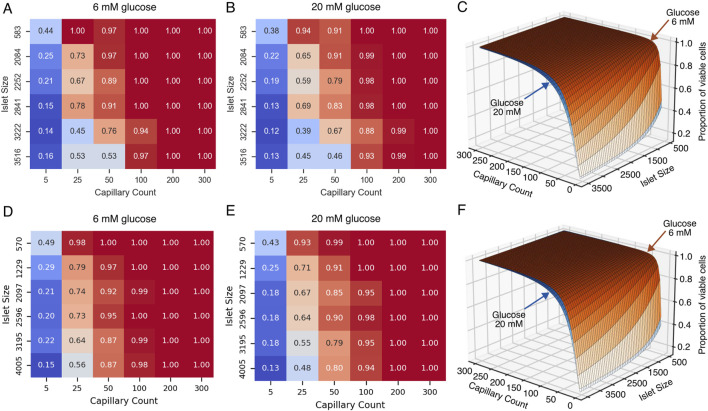
Effect of islet size and capillary count on the viability of islet cells under normal and high glucose conditions for human **(A–C)** and mice **(D–F)** islets. **(A,D)** Heatmap showing the proportion of viable cells in pancreatic islets under normal glucose conditions (6 mM), as a function of islet size (y-axis) and capillary count (x-axis). **(B)** Heatmap under high glucose conditions (20 mM). **(C,E)** Three-dimensional surface plot integrating data from panels **(A,B) (C)**, and **(D,E) (F)** illustrating how cell viability (z-axis) is influenced by both islet size (i.e., number of cells) and capillary count, with differential effects between normal (6 mM) and high (20 mM) glucose concentrations.

The heat maps shown in Figures 9A,B,D,E illustrate the relationship between glucose, islet size, capillary density, and proportion of viable cells (i.e., the summed proportion of functional and hypoxic cells) for G6 and G20 in both species. A clear gradient is observed from the lower left to the upper right of the heat maps in both glucose conditions, indicating that both islet size and vascular supply significantly influence cellular viability. These results show that adequate vascularization (≥100 capillaries or a vascular density of approximately 6% in human and mice islets) consistently results in near-complete viability (0.95–1.00) across all islet sizes. Even with minimal vascularization (5 capillaries), the proportion of viable cells ranged from 0.13–0.44 depending on islet size and glucose concentration, indicating that many cells remain viable but hypoxic under restricted vascular conditions. Interestingly, even larger islets showed relatively high viability proportions under limited vascular supply (e.g., 0.4–0.5 for islets with more than 3,000 cells with 25 capillaries).

### 3.3 Modeling cell viability as a nonlinear function of capillary number and islet size

To summarize our simulations results in an intuitive and practical way, we fitted a nonlinear regression model (Equation 3) to quantify the relationship between the number of viable total cells, number of capillaries, and total number of cells in the islet for both mice and humans. A graphical representation of the models of cell viability for human and mouse islets are shown in Figures 9C,F, respectively, where the position over the surface (i.e., proportion of viable cells) depend on both the capillary count and islet size in number of cells. The human model demonstrated robust performance with a mean squared error of 0.004 and a coefficient of determination (*R* ^2^) of 0.96 for both G6 and G20. Similarly, the mice model showed a mean squared error of 0.001 and *R* ^2^ of 0.99. The parameters of the fitted models are given in Table 4. The number of capillaries showed a cooperative effect, suggesting that moderate increases in the number of capillaries significantly enhanced viability. In contrast, the total cell number exhibited an inhibitory effect, reflecting the detrimental effect of islet size on cell viability.

**TABLE 4:** Parameters for the cell viability model under G6 and G20 conditions for human and mice islets. *V*_*min*_ and *V*_*max*_ represent the lower and upper bounds of viability proportion, respectively, *C*_*50*_ is the capillary number at which viability is half-maximal, *N*_*50*_ is the cell number at which viability is half-maximal, *n*_*C*_ and *n*_*N*_ are coefficients determining the steepness of the viability response to *N*_*cells*_ and *N*_*cap*_, respectively. All parameters are dimensionless. The goodness-of-fit parameter $R^{2}$ quantifies how well the model reproduces the data, with values closer to one indicating a better fit.

Human viability model							
*G* (mM)	*V*_*min*_	*V*_*max*_	C_50_	N_50_	*n*_*C*_	*n*_*N*_	*R* ^*2*^
6	0.13	1.00	6.82	669.16	1.92	−1.70	0.96
20	0.12	1.00	14.23	1,271.88	1.77	−1.60	0.96

Mice viability model							
*G* (mM)	*V*_*min*_	*V*_*max*_	C_50_	N_50_	*n*_*C*_	*n*_*N*_	*R* ^*2*^
6	0.14	1.00	9.29	969.42	2.02	−1.42	0.99
20	0.14	1.00	10.35	872.41	1.71	−1.42	0.99

The values of the estimated minimum viability (*V*_*min*_) reflect conditions of low vascularization where only a small proportion of cells remain in the viable range, either hypoxic or functional, while the maximum viability (*V*_*max*_ = 1.0) suggests that with an optimal number of capillaries and adequate islet sizes, maximal viability can be achieved. In summary, the proposed viability model effectively captures the nonlinear interaction between number of capillaries and islet size, providing a quantitative framework to predict viability across various experimental or transplant scenarios. An interactive tool where this model has been implemented in the associated repository (https://github.com/gjfelix/VascularizedIsletsModel).

## 4 Discussion

The vascular network of pancreatic islets plays a critical role in maintaining cell function, particularly in the secretion of hormones such as insulin, glucagon, and somatostatin. Emerging evidence suggests that the islet vascular system not only facilitates the delivery of nutrients and oxygen, but that its regulation also influences local blood flow, thereby affecting how islet cells sense nutrients and subsequently regulate hormone secretion. These findings underscore the importance of studying the islet vascular system to gain a deeper understanding of overall islet physiology.

In this study, we integrated experimental and computational approaches to develop a vascularized islet model. Our model introduces several novel elements and innovations to the field of pancreatic islet research. Previous multicellular models of pancreatic islets completely neglected islet vasculature and focused on other aspects such as inter-cellular communication and paracrine signaling (Félix-Martínez and Godínez-Fernández, 2023). Similarly, previous models of islet oxygenation including oxygen diffusion and consumption were developed to assess the viability of avascular islets, either naked or encapsulated (Félix-Martínez et al., 2024; Komatsu et al., 2017; Buchwald et al., 2015), even though islet microvasculature plays a key role in islet function. In contrast, the present model integrates, for the first time, capillary networks into multicellular islet models reconstructed from experimental data to better understand the delivery of oxygen to islet cells, allowing the model to provide a quantitative assessment of the degree of vascularization needed for proper oxygenation in both mouse and human islets, and therefore enabling species-specific insights through comparative analysis. In addition, the model considered multiple factors, including islet size and glucose concentration, to comprehensively understand how these variables impact oxygen gradients and cell viability at different levels of vascularization. Finally, the development of a user-friendly computational tool to estimate islet cell viability based on these factors represents a significant advancement in the field. This accessible tool allows researchers and clinicians to readily apply the model’s findings, addressing a common limitation of many existing islet models which often lack such practical implementation features. By facilitating the translation of computational results into easily interpretable outcomes, this tool enhances the utility of the model and its potential impact on research and clinical applications. Overall, by integrating vascular networks into computational models of pancreatic islets, this work lays the foundation for the development of more comprehensive models of pancreatic islets, thereby advancing the ability to simulate and understand islet function.

Using this model, we estimated that vascularization of ≥100 capillaries (vascular density >5.9%) is the minimum necessary for near-complete cell viability across all islet sizes according to oxygen availability. Experimental data from mice estimates that vascular density in islets ranges between 10% and 30%, with a mean value around 20%. In terms of the number of capillaries, these density values translate to 200–300 capillaries according to our model, where it predicts that >96% of cell will be functional at both 6 and 20 mM of glucose. Interestingly, a 4- to 5-fold lower vascular density has been reported in human islets compared with mice (Brissova et al., 2015; Cohrs et al., 2017). According to our simulated human islets, a 5% vascular density is reached near a count of 100 capillaries where, as stated before, a high viability percentage is possible both at low and high glucose. However, these anatomical differences mean that findings from rodent islets may not directly translate to human islets, particularly regarding the threshold for hypoxia and optimal vascular density required to maintain islet function. Therefore, computational models based on real human islet architectures are essential for accurately predicting oxygen gradients and hypoxic risk. Notably, our model predicts that for both mice and humans, the vascular density observed experimentally is compatible with the presence of functional cells to maintain islet function. However, according to our simulations, a vascular density lower than 5% (i.e., <100 capillaries) would compromise the functional status of cells.

Changes in the vascular system within pancreatic islets have been associated with diabetes and aging. Chen et al. (2021) reported changes in vascular density in human pancreatic islets depending on age or diabetes status. Specifically, they found a significant age-related decline in vessel density and artery numbers in the human endocrine pancreas, as demonstrated by 3D imaging and quantitative analysis of young (<20 years) *versus* aged (>70 years) tissues, accompanied by reduced expression of key endothelial and angiogenic markers. The decline in vessel density is particularly important because their loss with age may contribute to impaired β-cell maintenance and function which, according to our results, could be partly produced by the lack of proper oxygenation in certain regions of the islets. These findings have clinical implications, suggesting that strategies to preserve or restore islet vascularization could be tested *in silico* aiming to maintain β-cell mass and function in ageing. Along with the changes in vascular density, there are also reports of fibrosis and inflammation in blood vessels of both rodent and human islets (Almaça et al., 2014), which could impair islet function further.

Alterations in the vasculature of human islets have been reported in type 1 and type 2 diabetes, with research showing significant remodeling characterized by changes in capillary density, diameter, and structure (Gonçalves et al., 2024). In type 1 diabetes, reports by Canzano et al. (2019) and Granlund et al. (2022) showed increased vessel density but reduced vessel diameter. More recently, Richardson et al. (2023) showed that capillary density from human is increased in recent-onset T1D compared to non-diabetic and longstanding T1D, while Wang et al. (2019) reported a reduction in vascular density depending on the region of the pancreas and the duration of T1D. In type 2 diabetes, islets exhibit increased capillary density, thickening, and fragmentation, often associated with amyloid deposition, but these changes do not necessarily translate to improved oxygenation and may even exacerbate hypoxia due to disrupted vessel integrity and function (Brissova et al., 2015; Aplin et al., 2024). Moreover, both forms of diabetes are also marked by microvascular abnormalities, loss of pericyte coverage, increased vascular permeability, and excessive extracellular matrix deposition, all of which can further compromise oxygen and nutrient delivery to endocrine cells (Gonçalves et al., 2024; Aplin et al., 2024). These pathological changes suggest that the optimal vascular density for preventing hypoxia in healthy islets may not apply to diabetic islets, emphasizing the need for computational models, such as the one presented in this work, to account for vessel density, spatial distribution, and functional capacity when predicting islet oxygenation and guiding therapeutic interventions.

Our model has direct application for designing tissue engineering and regenerative therapy strategies, where balancing vascularization and cell density is crucial for maximizing tissue survival and functionality. Vascularization strategies are critical for optimizing the oxygenation of islet grafts immediately following transplantation (Li et al., 2025; Bender et al., 2024). By engineering the transplantation site with enhanced vascular networks through approaches such as prevascularized tissues, vascularizing devices, or co-transplantation of angiogenic factors, a microvascular supply can be established prior to graft implantation. This vascular support is essential, as it reduces the diffusion distance for oxygen and mitigates the risk of hypoxia-induced graft loss before full revascularization occurs. However, the effectiveness of these strategies depends on a deep understanding of the degree of vascularization required to meet the metabolic demands of transplanted islets. If the density of blood vessels is inadequate, the oxygen supply will be less than optimal, putting the survival of the graft at risk. Conversely, excessive vascularization may not further increase tissue oxygenation once the islet oxygen demand is met. Therefore, quantifying and designing vascularized structures to match the oxygen requirements of the graft is fundamental for the success of islet transplantation and the development of successful oxygenation strategies. In this regard, our model of viability could serve as a guide for the design of vascularizing devices or strategies where morphological, structural, and functional variables are considered beforehand.

Beyond vascularization, unresolved questions remain about how islet architecture, including endocrine cell proportions and organization, innervation, and polarity, impacts function in health and disease. Future studies integrating these factors with vascular dynamics could further clarify their collective role in islet viability and function. Moreover, other aspects related to islet vasculature are current topics of active research, such as the functional role of blood flow and its direction (i.e., pole to pole, periphery to center or center to periphery) (Diez et al., 2017; Ballian and Brunicardi, 2007), the role of pericytes as gatekeepers of blood flow within islets (Gonçalves and Almaça, 2021; Gonçalves et al., 2024) and microvascular alterations in diabetes (Canzano et al., 2019; Gonçalves et al., 2024; Wang et al., 2019).

The present model has limitations that should be acknowledged. First, it employs a simplified vascular geometry, a choice that might not fully capture the complex branching and tortuosity of real islet vasculature or small-scale details such as fenestrations. Nevertheless, this simplification made it possible to explore a wide range of vascular densities across species in a controlled setting, while reproducing experimentally observed capillary radii distributions. Second, blood flow dynamics, hemodynamic details, and directional effects, which could influence the oxygen distribution patterns, were not included. However, their exclusion allowed for a more focused study of diffusion-based oxygen transport, highlighting the principles underlying oxygen gradients within mice and human islets. Finally, hormone secretion and interactions between different cell types and paracrine signaling effects were omitted to maintain a clear focus on oxygen availability as the main variable, facilitating an understanding of its direct influence before introducing further complexity. Despite these constraints, the model provides a valuable and controlled framework for studying the oxygen distribution within pancreatic islets, and to assess the impact of the degree of vascularization on the viability of islet cells. Further versions of the model integrating these elements could enhance our ability to predict and manage diabetes-related complications, as well as inform the development of more effective therapeutic strategies targeting islet vasculature.

In summary, our study highlights the critical relationship between islet vascularization, oxygenation, and cell viability, with potential implications for aging, diabetes, and islet transplantation. While our model advances the understanding of vascular requirements for islet survival, key questions remain regarding blood flow dynamics, pericyte regulation, and adaptive changes in disease. Future work should integrate these factors to refine predictive models and develop targeted vascular therapies. By bridging computational and experimental approaches, we move closer to preserving functional islet mass in metabolic disorders and improving outcomes in regenerative medicine.

## Data Availability

The datasets presented in this study can be found in the repository associated to the article (https://github.com/gjfelix/VascularizedIsletsModel).
